# Intake of Alpha-Linolenic Acid-Rich *Perilla frutescens* Leaf Powder Decreases Home Blood Pressure and Serum Oxidized Low-Density Lipoprotein in Japanese Adults

**DOI:** 10.3390/molecules25092099

**Published:** 2020-04-30

**Authors:** Michio Hashimoto, Yoko Tanabe, Shahdat Hossain, Kentaro Matsuzaki, Miho Ohno, Setsushi Kato, Masanori Katakura, Osamu Shido

**Affiliations:** 1 Department of Environmental Physiology, Faculty of Medicine, Shimane University, Izumo 693-8501, Shimane, Japan; tanabey@med.shimane-u.ac.jp (Y.T.); matuzaki@med.shimane-u.ac.jp (K.M.); mkatakur@josai.ac.jp (M.K.); o-shido@med.shimane-u.ac.jp (O.S.); 2 Department of Biochemistry and Molecular Biology, Jahangirnagar University, Savar, Dhaka 1342, Bangladesh; shahdat@juniv.edu; 3 Kato Hospital, Jinjukai Healthcare Corporation, Kawamoto, Shimane 696-0001, Japan; ohno@k-jinju.or.jp (M.O.); katosetsu@k-jinju.or.jp (S.K.)

**Keywords:** oxidized low-density lipoprotein, *Perilla frutescens*, atherosclerosis, biological antioxidative potential, systolic blood pressure, α-linolenic acid

## Abstract

Oxidized low-density lipoprotein (Ox-LDL) is known to be highly atherogenic. Thus, decreasing the blood levels of Ox-LDL through dietary means is an important approach to reduce cardiovascular events in high-risk individuals. In this randomized placebo-controlled human interventional trial, we aimed to evaluate whether *Perilla frutescens* leaf powder (PLP) ameliorates Ox-LDL and home blood pressure, along with its biological antioxidant potential. Healthy Japanese volunteers aged 30–60 years (*n* = 60) were randomized to PLP and placebo groups. The PLP group consumed PLP dried using a microwave under reduced pressure, and the placebo group consumed pectin fiber daily for 6 months. Home blood pressure, serum biochemical parameters, and fatty acid profiles of erythrocyte plasma membranes were analyzed. Plasma Ox-LDL levels significantly decreased in the PLP group but not in the placebo group. Mean changes in the biological antioxidant potential and alpha-linolenic acid levels in the erythrocyte plasma membrane were significantly increased in the PLP group than in the placebo group. In subjects with prehypertension (systolic blood pressure [SBP] ≥ 120 mmHg), the mean reduction in morning or nocturnal SBP was significantly greater in the PLP group than in the placebo group. Thus, PLP intake may be an effective intervention to prevent cardiovascular diseases.

## 1. Introduction

The seed of *Perilla frutescens* is used to derive vegetable oil, namely perilla oil. In addition to its seed oil, the leaves of *P. frutescens* have numerous applications, such as a popular garnish in foods in Japan, China, and Korea [1,2]; as a preservative; and for medicinal purposes, such as in the treatment of colds, food poisoning [3], food allergy [4], and depression [5]. Red-colored perilla leaves are also frequently used as a food colorant owing to the presence of anthocyanins and other related compounds, which themselves have free radical-scavenging potential [6] and anti-lipid peroxidation effects [7]. Furthermore, phenolic compounds, such as rosmarinic acid, luteolin, apigenin, and α-linolenic acid (ALA), are abundant in the leaves of perilla and have antiallergic, anti-inflammatory [8,9], and neuroprotective effects [10], indicating that perilla leaves mimic many of the demonstrated effects of perilla oil. Notably, perilla oil is the richest known source of ALA [11], an omega-3 polyunsaturated fatty acid molecule (ω-3 ALA, C18:3), which confers the oil with antithrombotic [12], antiarrhythmic [13], antiatherosclerotic [14], anti-inflammatory [15], and neuroprotective [16] effects. We previously reported that perilla leaf powder (PLP), microwave dried under reduced pressure, reduced the risk of metabolic syndrome in the SHR.Cg-*Lepr^cp^*/NDmcr (SHR-cp) rat model [17]. The changes in blood pressure (BP) from baseline to week 14 decreased significantly in the PLP-fed SHR-cp rats compared with those of the control group. Plasma triglyceride and total cholesterol levels also decreased significantly, concurrent with increases in the plasma levels of ALA and total omega-3 fatty acids. Plasma lipid peroxide levels also decreased significantly in the PLP-fed metabolic model rats. These results suggest that PLP could have potent antioxidative, antihypertensive, and antihyperlipidemic effects, resulting in an overall preventive effect against cardiovascular diseases.

Oxidative stress plays a major role in the pathogenesis of metabolic syndrome [18], cardiovascular disease [19], and aging and age-related neurodegenerative diseases [20,21]. Several studies have reported that the oxidation of low-density lipoprotein (LDL) increases in cardiovascular diseases, suggesting the level of oxidized LDL (Ox-LDL) as a useful diagnostic and prognostic marker for cardiovascular diseases [22,23,24]. The oxidation of LDL is a complex process involving oxidative changes of both proteins and lipids that form complex products [25,26]. Ox-LDL is then scavenged by macrophages [27], which in turn transform into foam cells that accumulate to contribute to atherosclerosis [28]. Epidemiological studies suggest that the risk of cardiovascular diseases and mental and neurological disorders can be reduced by increasing the consumption of fruits and vegetables [29,30,31]. Therefore, it is commonly believed that dietary fruit and vegetable intake is beneficial in preventing disease onset. Given the potent biological effects of PLP described above, we conducted a randomized placebo-controlled human interventional trial over 6 months to examine the effect of dietary PLP intake on Ox-LDL, biological antioxidant potential (BAP), home BP, and other blood parameters in Japanese adults. These findings can provide a basis for PLP dietary supplementation as a measure to prevent cardiovascular diseases in high-risk individuals.

## 2. Results

### 2.1. Baseline Characteristics

This study included a total of 30 subjects, who were randomly allocated to the PLP group and placebo (pectin fiber) group. All baseline characteristics of the subjects are shown in Table 1 and Table 2. There were no significant intergroup differences in sex, age, height, body weight, body fat, body mass index, waist circumference, and BP (Table 1), and in serum biochemical and hematological parameters (Table 2) between the two groups.

The study was performed for a total of 6 months; five participants in the placebo group and two participants in the PLP group dropped out (Table 1). All participants joined the study on a volunteer basis and were free to withdraw at any time. The seven participants who dropped out did not become ill by participating in this study but rather withdrew for personal reasons, including the inconvenience of taking home BP measurements 2–3 days a week at waking and bedtime and having to consume PLP/placebo daily. 

### 2.2. Nutritional Intake

There were no significant differences between the placebo and PLP groups in the mean dietary nutritional intake for 6 months, based on responses to the self-administered dietary history questionnaire for Japanese adults (BDHQ) (data not shown). No side effects that disturbed the daily life of the participants (such as allergic reactions, palpitations, and irritation in the stomach) were noted in either group.

### 2.3. Effect of PLP Intake on Body Weight, Waist Circumference, and Body Composition

PLP intake for 6 months did not significantly affect the body weight, body mass index, waist circumference, and fat mass compared with those in the placebo group (Table 1).

### 2.4. Effect of PLP Intake on Blood Biochemical and Hematological Parameters 

Except for the individuals who withdrew from the study, the participants showed high adherence to the study protocol for 6 months (92.1% ± 2.5%). Data obtained from the general questionnaire assessing lifestyle habits and medical history did not show differences from baseline and the end of the trial (6 months). There were no differences in serum biochemical and hematological parameters between the two groups at 6 months (Table 2), indicating that PLP intake did not affect hepatic and renal functions, blood lipid metabolism, or hematopoietic function. These results suggested the potential safety of PLP intake for 6 months.

### 2.5. Effect of PLP Intake on Home BP

**Figure 1:**
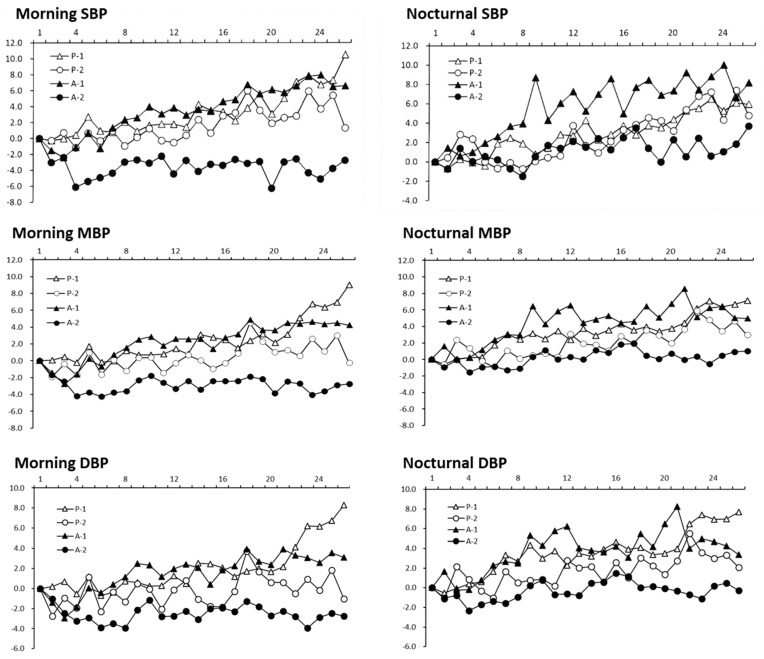
Effects of *Perilla frutescens* leaf powder (PLP) intake on mean changes in home systolic, mean, and diastolic blood pressure. Self-measurement of blood pressure at home was performed daily with an automatic upper-arm device, after waking up (morning systolic blood pressure [SBP], morning mean blood pressure [MBP], and morning diastolic blood pressure [DBP]) and before going to bed (nocturnal SBP, nocturnal MBP, and nocturnal DBP). P-1 (∆), placebo-treated subjects who ingested pectin fiber with SBP < 120 mmHg (*n* = 7); P-2 (○), placebo-treated subjects who ingested pectin fiber with SBP ≥ 120 mmHg (*n* = 5); A-1 (▲), subjects who ingested PLP and had SBP < 120 mmHg (*n* = 10); A-2 (●), subjects who ingested PLP and had SBP ≥ 120 mmHg (*n* = 8). Each symbol represents the values averaged for 1 week. Data are shown as the mean changes in BP levels (mmHg) from baseline to the estimated week.

At month 6, there were no significant differences in systolic BP (SBP), mean BP (MBP), and diastolic BP (DBP) between the placebo and PLP groups (Table 1). Some participants did not measure home BP during the study; thus, complete home BP data for 6 months were only available for 30 participants (*n* = 12 in the placebo group and *n* = 18 in the PLP group). Randomized two-factor (week and group) analysis of variance (ANOVA) showed no significant main effects of PLP intake on home SBP, MBP, and DBP both among the weeks of measurement and between groups (*P* values of the week × group interaction were 0.662 for morning SBP, 0.622 for morning MBP, 0.272 for morning DBP, 0.607 for nocturnal SBP, 0.712 for nocturnal MBP, and 0.236 for nocturnal DBP; data not shown). Therefore, home BP values were assessed by the change from baseline in each experimental group separately. When subjects were divided into two groups according to a home SBP ≥ 120 mmHg and < 120 mmHg at month 6, randomized two-factor ANOVA revealed significant main effects of PLP intake for both weeks of measurement and groups (Figure 1). There were also significant week × group interactions with regard to the morning SBP (F = 1.96, *P* < 0.0001), morning MBP (F = 1.34, *P* < 0.0006), morning DBP (F = 1.75, *P* = 0.0002), and nocturnal SBP (F = 1.40, *P* = 0.0183) (Figure 1). However, there was no significant week × group interaction with regard to nocturnal MBP (F = 1.72, *P* = 0.067) and nocturnal DBP (F = 1.23, *P* = 0.0986; Figure 1).

Subgroup analyses (Table 3) of the morning and nocturnal BP showed a significant effect of PLP intake on the mean changes in the BP of subjects with home SBP ≥ 120 mmHg (significant week × group interaction for morning SBP, *P* = 0.005 and nocturnal SBP, *P* = 0.016). These analyses demonstrated that the mean changes in the morning and nocturnal SBP of the subjects with home SBP ≥ 120 mmHg at month 6 were significantly lower in the PLP group than in the placebo group, suggesting that PLP intake prevents an age-related increase in SBP.

### 2.6. Effect of PLP Intake on the Fatty Acid Profile of Erythrocyte Plasma Membranes

At baseline, there were no significant differences in the levels of any fatty acid between the placebo and PLP groups. At month 6, the concentration of ALA was significantly higher in the PLP group than in the placebo group, whereas no other fatty acids were affected by PLP intake (Table 4). This increase in the mean ALA level of the erythrocyte plasma membrane further supported the consumption compliance in the PLP group.

### 2.7. Effect of PLP Powder Intake on Serum Ox-LDL 

**Figure 2:**
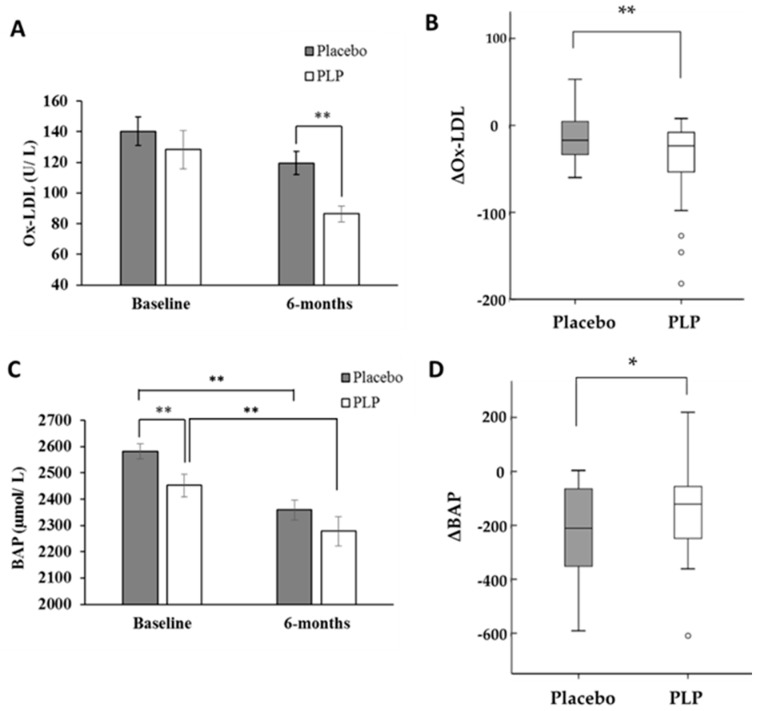
Changes of serum-oxidized low-density lipoprotein (Ox-LDL) (**A**,**B**) and biological antioxidant potential (BAP) (**C**,**D**) in the placebo and *Perilla frutescens* leaf powder (PLP) groups. (**B**,**D**) indicate the mean changes (∆) in Ox-LDL and BAP levels from baseline to month 6 in each group. Vertical bars represent the standard error of mean (**A**,**C**) or interquartile range (**B**,**D**). ** *P* < 0.05, * 0.05 < *P* < 0.1.

The effects of PLP intake on serum Ox-LDL levels and BAP are shown in Figure 2. At baseline, the serum Ox-LDL levels were not significantly different between the placebo and PLP groups (Figure 2A). However, 6 months of PLP and placebo intake decreased the serum concentrations of Ox-LDL from baseline by 41.9 and 20.7 U/L, respectively, representing significant intergroup differences in the levels of Ox-LDL at the end of the intervention (Figure 2B). 

**Figure 3:**
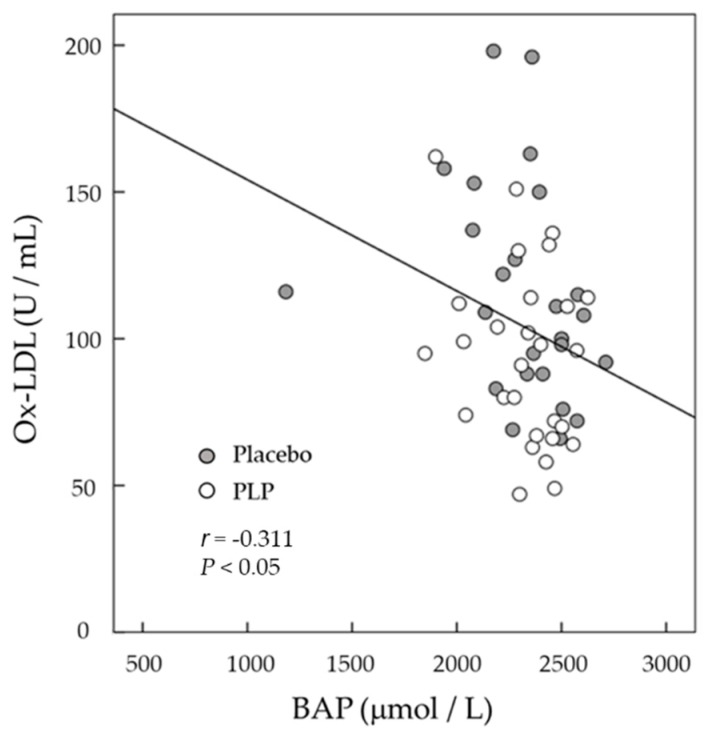
Scatter plots of the relationship between serum-oxidized low-density lipoprotein (Ox-LDL) and biological antioxidant potential (BAP) levels at 6 months. (○), placebo group; (●) perilla leaf powder (PLP) intervention group.

The BAP test measures the ability for reducing ferric (Fe^3+^) to ferrous (Fe^2+^) ions, according to the same principle of the ferric reducing antioxidant power (FRAP) assay [32]. In addition, serum BAP levels were reported to be significantly associated with serum Ox-LDL levels in humans [33,34]. At baseline, the serum BAP level was significantly higher in the placebo group than in the PLP group (Figure 2C), and the PLP and placebo treatments significantly decreased the BAP from baseline by 174 and by 223 μM, respectively, showing a tendency toward a significant difference (0.05 < *P* < 0.1) between the two groups (Figure 2D). Moreover, serum BAP levels were significantly correlated with Ox-LDL levels at 6 months (Figure 3; *r* = −0.311, *P* < 0.05), whereas no significant correlation was observed at baseline (*P* > 0.05).

## 3. Discussion

In the present study, we sought to determine the effects of PLP intake on Ox-LDL, home BP, and BAP in Japanese adults. PLP intake significantly reduced serum Ox-LDL levels and home SBP among subjects with a home SBP ≥ 120 mmHg compared with those of the placebo group. There were no effects of PLP intake on hepatic and renal function biomarkers, body weight, or the incidence of adverse events based on clinical interviews and daybooks, demonstrating the safety of the long-term intake of PLP.

Ox-LDL is internalized by macrophages and endothelial cells via specific receptors, leading to endothelial dysfunction and foam cell formation. Therefore, the targeting of Ox-LDL has been proposed as a potentially promising approach to prevent atherosclerosis. In this study, we found that PLP intake lowered plasma Ox-LDL levels; thus, further assessment of whether PLP reduces lipid peroxidation would be of high importance to evaluate its overall antioxidative potential. A previous study showed that oral administration of PLP significantly decreased the plasma lipid peroxidation levels in SHR-cp rats, suggesting the antilipid peroxidation effects of PLP, which may be related to some of its components, such as rosmarinic acid and lutein [17].

PLP contains polyphenols, including cinnamic acid derivatives (e.g., caffeic acid and rosmarinic acid), flavonoids, and anthocyanins [10], as well as certain proteins. Saita et al. [35] reported that PLP could scavenge 2,2-diphenyl-1-picrylhydrazyl (DPPH) free radicals in vitro, and induced the production of antioxidative enzymes, including catalase and superoxide dismutase, in human umbilical vein endothelial cells, thereby inhibiting the endothelial cell-induced oxidation of LDL. Our findings are also consistent with those of Ruel et al. [36] and Egert et al. [37], who showed that flavonoid-rich cranberry juice or purified polyphenols, such as quercetin, significantly decreased Ox-LDL concentrations in humans. Thus, it is possible that the polyphenols present in PLP inhibited the oxidation of LDL, leading to the observed decrease in the Ox-LDL serum concentration in the PLP group in the present study. Moreover, the decrease in serum Ox-LDL levels in the PLP group after the 6-month intervention was accompanied by a decrease in serum BAP levels. However, the change in BAP levels from baseline to 6 months in the PLP group was relatively higher than that of the placebo group. BAP is a widely used biomarker of oxidative stress, as it can be conveniently measured in large-scale epidemiological research reflecting the total antioxidant status of the serum [38]. In the present study, serum BAP levels were significantly negatively associated with Ox-LDL levels at month 6. Although the detailed mechanism is unclear, our results suggest that PLP may at least partly act as an antioxidant in vivo and decrease the levels of Ox-LDL. Further research, including measurement of the reactive oxygen species and/or FRAP levels, is required to establish a causal link between serum Ox-LDL reduction and the antioxidant effects of PLP in human subjects.

Notably, we found that PLP intake significantly increased the levels of ALA in the erythrocyte plasma membranes, whereas the levels of other ω-3 polyunsaturated fatty acids, including eicosapentaenoic acid and docosahexaenoic acid, were not altered. ALA has been shown to exhibit antihypertensive effects in human subjects [39,40] without any peroxidative effects [40]. It also had a preventive effect against strokes and ischemia in humans [41], and high intake of ALA decreased the prevalence of carotid plaques [42]. Zhao et al. [43] reported that ALA might decrease cardiovascular disease risk by inhibiting vascular endothelial cell activation. Therefore, the PLP-induced increase in ALA of the endothelial membrane might have influenced the vascular endothelial beds to affect the blood vessel tone and ultimately reduce BP in the present study.

Morning hypertension (i.e., higher BP in the early morning than in the evening) has recently attracted substantial attention as a potential link to understand the relationship between BP levels in the early morning and cardiovascular risk. Cases of morning hypertension are classified into two types: The “morning-surge” type, characterized by a marked increase in BP in the early morning, and the “nocturnal-hypertension” type, characterized by high BP that persists from nighttime until early morning. PLP intake significantly decreased home SBP in prehypertensive subjects (SBP > 120 mmHg) both in the morning and at night. To our knowledge, no human study has examined the effect of PLP supplementation on BP. However, several studies have shown that purified polyphenols, such as quercetin, decreased BP in subjects with stage-1 hypertension but not in those with prehypertension, suggesting that a certain degree of hypertension is required for quercetin to exert a BP-lowering effect [44]. In addition, as PLP contains more than approximately 20% protein, some other unknown antihypertensive substances may be involved in the observed effects. Since our study did not include subjects with stage-1 hypertension, it is difficult to infer the effect of PLP on BP in that condition. In contrast, PLP intake clearly ameliorated the prehypertension in our subjects with SBP ≥120 mmHg. The circulating Ox-LDL level is elevated in hypertension [45]; however, the cause–effect relationship needs to be clarified. We hypothesize that PLP-induced enhancements in endothelial functions might be the underlying mechanism by which PLP ameliorates SBP. Therefore, supplementation with PLP could be an effective strategy to reduce cardiovascular risk. 

The main limitation of this study is that it was an interventional study in healthy subjects, aimed at finding an effective agent for the primary prevention of cardiovascular disease. Furthermore, this study included a relatively small number of subjects from a localized sample population (a cohort of Japanese individuals from one town), which limits the ability to generalize the findings. Moreover, we lacked information on the genetic background of the subjects and did not continue follow-up after termination of the study. In addition, the changes in home blood pressure were defined as the difference between blood pressure and the baseline in order to achieve statistical differences, since there were no significant differences in morning MBP or nocturnal MBP between the placebo and PLP groups.

## 4. Materials and Methods 

### 4.1. Subjects 

This 6-month randomized placebo-controlled study was conducted among healthy 30- to 60-year-old adults living in Shimane Prefecture of Japan. Volunteers underwent anthropometric and blood biochemistry tests. Individuals were excluded if there was any evidence of a medical disorder, including renal, respiratory, cardiac, or hepatic disease; diabetes mellitus; and endocrine, metabolic, or hematological disturbances. Individuals using any psychotropic drug/supplement that might significantly influence the outcomes of the study were also excluded. 

The subjects were assigned to the placebo (*n* = 30) or PLP (*n* = 30) group. Those in the placebo group consumed pectin fiber (9 g/day; GENU pectin type 121-J slow set, Sansho Co., Ltd, Osaka, Japan) daily for 6 months, and those in the PLP group consumed PLP (9 g/day; O-san farm Co., Kawamoto, Japan) daily for 6 months. The nutrient compositions of pectin fiber and PLP are listed in Table 5 and Table 6, respectively. This study was conducted according to the principles of the Declaration of Helsinki and Good Clinical Practice, and was approved by the Ethics Committee of Kato Hospital (approval number 2011-006). This interventional study was performed during 2011–2012. All participants provided written informed consent before participation. Monthly health checks and consumption compliance were undertaken to encourage adherence to research protocols. 

### 4.2. Anthropometry, Body Composition, and Home BP Measurements 

Body weight, height, and waist circumference were measured by hospital nurses. Body composition was determined by bioelectrical impedance analysis (WB-150, TANITA Co., Tokyo, Japan). To investigate the effects of PLP intake on health status and lifestyle during the trials, a general questionnaire was administered, including lifestyle questions and those related to medical history. Dietary intake was estimated using a brief self-administered dietary history questionnaire (BDHQ) for Japanese adults. 

Self-measurement of home BP was performed with an upper-arm device (HEM-1010, OMRON Co., Tokyo, Japan) two times per day; morning BP was measured within 1 h after waking up and nocturnal BP was measured before going to the bed at least 3 days per week for 6 months. The BP was measured without meals or exercise. In this study, home BP was evaluated as the change from baseline in each experimental group.

### 4.3. Blood Sampling 

Before ingestion (baseline) and after 6 months of the intervention, blood samples were obtained in the morning or afternoon after ascertaining that the participants had not had breakfast or lunch. Blood samples were separated into serum, plasma, and erythrocyte (red blood cell [RBC]) aliquots. After measuring the clinical parameters, the remaining serum and RBCs were stored at −80 °C until analysis. To monitor the fatty acid profile in the erythrocyte plasma membranes, whole RBCs were washed until they appeared white, as previously described [46].

### 4.4. Clinical Safety Parameters

The following fasting blood sample parameters were evaluated. Hematological parameters included the white blood cell count, red blood cell count, hemoglobin, hematocrit, platelets, mean corpuscular volume, mean corpuscular hemoglobin, and mean corpuscular hemoglobin concentration using a commercial clinical laboratory service (SRL Inc., Tokyo, Japan). Biochemical analyses included aspartate aminotransferase, alanine aminotransferase, gamma-glutamyl transpeptidase, albumin, total cholesterol, triglycerides, blood urea nitrogen, creatinine, blood sugar, high-density lipoprotein cholesterol, and LDL cholesterol using an automatic analyzer (BiOLis 24; Tokyo Boeki Medical System, Tokyo, Japan). Hemoglobin A1c was measured with a kit from TFB (Tokyo, Japan). 

### 4.5. Fatty Acid Composition

The fatty acid composition of the erythrocyte plasma membranes was determined using gas chromatography as previously described [47]. In brief, 100 μL of erythrocytes were suspended by vigorous mixing in 1.2 mL of a 4 mM ethylenediaminetetraacetic acid solution containing 0.005% 2,6-di-t-butyl-4-methylphenol (BHT) and centrifuged at 10,000× *g* for 15 min at 4 °C. After the supernatant was discarded, the resultant pellet was resuspended by vigorous mixing in 1 mL of Dulbecco’s phosphate-buffered saline (PBS)(−) containing 0.005% BHT and centrifuged at 10,000 rpm for 15 min at 4 °C. This procedure was repeated once. The final pellet was homogenized in 250 μL of Dulbecco’s PBS(−) containing 0.5% saponin and 0.005% BHT using an ultrasonic homogenizer (Bioruptor; Cosmobio Co., Tokyo, Japan), which was subjected to the measurement of fatty acids and proteins. The fatty acid concentrations in the erythrocyte plasma membrane suspension were measured by capillary gas chromatography of the corresponding methyl esters prepared by transesterification with acetyl chloride.

### 4.6. BAP Assay

BAP was measured using a free radical elective evaluator (FREE Carpe Diem) that included a spectrophotometric device reader, and measurement kits (BAP test) (Wismerll Co. Ltd., Tokyo, Japan) were optimized to the FREE Carpe Diem System according to the manufacturer’s protocol. The BAP assay is based on the ability of the sample (serum in this case) to reduce Fe^3+^ ions bound to a chromogenic substrate (ammonium thiocyanate) to Fe^2+^ ions. The substrate-containing solution becomes decolorized according to the reducing power of the sample.

### 4.7. Serum Ox-LDL Assay

Serum Ox-LDL levels were measured by a commercial clinical laboratory (SRL Inc., Tokyo, Japan).

### 4.8. Statistical Analysis

Prior to statistical analyses, the Shapiro–Wilk test was performed to assess the distribution of the data. Home BP data were analyzed using two-way (week and group) repeated-measures ANOVA, and the fatty acid profile of the erythrocyte plasma membrane, blood biochemical and hematological parameters, and serum Ox-LDL and BAP levels were analyzed for intergroup differences by one-way ANOVA. ANOVA was followed by Fisher’s protected least-significant differences test for post-hoc comparisons. These results are expressed as the mean and standard error of the mean (SEM). 

The correlation between serum BAP and Ox-LDL levels was evaluated by Pearson’s correlation coefficient. ∆Ox-LDL and ∆BAP from baseline to month 6 in the placebo and PLP groups was analyzed by the Mann–Whitney U test, and the results are presented as the median value and interquartile range. GB-STAT 6.5.4 (Dynamic Microsystems, Inc., Silver Spring, MD, USA) and Stat View 4.01 (Mind Vision Software, Abacus Concepts, Inc., Berkeley, CA, USA) were used for statistical analyses. Statistical significance was set at *P* < 0.05.

## 5. Conclusions

PLP intake is safe and has an antioxidant effect in vivo. PLP also reduced home SBP in adult subjects with prehypertension (SBP ≥ 120 mmHg). However, further investigation is required to determine the exact mechanism of action of PLP in a large number of human studies.

## Figures and Tables

**Table 1:** Body composition and blood pressure of subjects.

	Baseline		6 Months	
	Placebo	PLP	Placebo	PLP
	(n = 30)	(n = 30)	(n = 25)	(n = 28)
Gender (male/females)	15/15	15/15	11/14	15/13
Age (years)	46.7 ± 1.6	47.4 ± 1.5	47.9 ± 1.9	48.0 ± 1.7
Height (cm)	161.9 ± 1.5	161.2 ± 1.5	161.4 ± 1.8	162.3 ± 1.8
Body weight (kg)	64.1 ± 2.4	60.6 ± 1.9	63.1 ± 3.0	64.2 ± 1.7
Body Fat (%)	28.8 ± 1.1	27.2 ± 1.4	29.4 ± 1.2	28.1 ± 1.6
Body mass index (kg/m^2^)	24.4 ± 0.7	23.5 ± 0.6	24.1 ± 0.8	24.5 ± 0.5
Waist circumference (cm)	84.6 ± 1.7	82.7 ± 1.4	84.6 ± 2.1	85.6 ± 1.4
Blood pressure				
Systolic BP (mmHg)	132.8 ± 2.9	130.3 ± 4.1	136.7 ± 4.0	136.8 ± 4.1
Mean BP (mmHg)	100.0 ± 2.5	96.8 ± 3.3	104.3 ± 3.6	103.3 ± 3.0
Diastolic BP (mmHg)	83.8 ± 2.1	80.1 ± 2.6	88.1 ± 2.7	86.5 ± 2.9

Values are expressed as the standard error of mean (SEM). Placebo: the placebo group; PLP, *Perilla frutescens* leaf powder-intake group; BP: Blood pressure.

**Table 2:** Serum biochemical and hematological parameters of subjects.

	Baseline		6 months	
	Placebo	PLP	Placebo	PLP
	(n = 30)	(n = 30)	(n = 25)	(n = 28)
Serum biochemical parameters				
AST (U/L)	23.2 ± 1.2	24.3 ± 1.1	24.4 ± 1.3	27.8 ± 1.8
ALT (U/L)	27.3 ± 2.9	22.4 ± 1.8	26.7 ± 3.1	26.8 ± 2.6
γ-GTP (IU/L)	49.6 ± 9.1	50.4 ± 10.9	40.3 ± 6.0	40.7 ± 7.4
Albumin (g/dL)	4.8 ± 0.03	4.7 ± 0.05	4.7 ± 0.05	4.6 ± 0.05
T-CHO (mg/dL)	237.1 ± 7.4	221.0 ± 7.4	238.7 ± 8.3	225.7 ± 6.3
Triglyceride (mg/dL)	120.6 ± 16.2	118.4 ± 12.8	108.7 ± 12.6	116.6 ± 13.9
BUN (mg/dL)	17.2 ± 0.6	17.2 ± 0.7	13.1 ± 0.5	14.2 ± 0.7
Creatinine (mg/dL)	0.74 ± 0.02	0.75 ± 0.03	0.72 ± 0.03	0.78 ± 0.03
Blood sugar (mg/dL)	105.9 ± 3.7	99.8 ± 1.3	99.7 ± 2.7	94.5 ± 2.5
HDL-C (mg/dL)	71.6 ± 3.5	76.0 ± 3.1	73.5 ± 4.2	76.6 ± 4.1
LDL-C (mg/dL)	152.1 ± 6.0	134.0 ± 6.2	142.0 ± 5.8	130.8 ± 5.8
HbA1c (%)	5.0 ± 0.08	4.8 ± 0.04	5.1 ± 0.07	5.0 ± 0.05
Hematological parameters				
WBC (×10^3^/μL)	5.8 ± 0.3	6.2 ± 0.3	5.7 ± 0.3	6.0 ± 0.3
RBC (×10^4^/μL)	482.4 ± 7.6	475.8 ± 6.9	472.2 ± 8.7	473.0 ± 8.2
Hemoglobin (g/dL)	14.4 ± 0.3	14.3 ± 0.2	14.1 ± 0.4	14.2 ± 0.3
Hematocrit (%)	43.0 ± 0.7	42.7 ± 0.6	42.4 ± 1.0	42.6 ± 0.7
Platelets (×10^4^/μL)	26.3 ± 1.1	25.4 ± 1.1	26.4 ± 1.6	25.0 ± 1.1
MCV (fL)	89.3 ± 0.9	89.9 ± 0.8	89.7 ± 1.2	90.1 ± 0.9
MCH (pg)	29.9 ± 0.4	30.1 ± 0.3	29.8 ± 0.6	30.1 ± 0.4
MCHC (%)	33.4 ± 0.2	33.4 ± 0.1	33.1 ± 0.3	33.4 ± 0.2

Values are expressed as the standard error of mean (SEM). Placebo: the placebo group, PLP, *Perilla frutescens* leaf powder-intake group. ALT; alanine aminotransferase, AST; aspartate aminotransferase, BUN; blood urea nitrogen, γ-GTP; γ-glutamyltranspeptitase, HbA1c; hemoglobin A1c, HDL-C; high-density lipoprotein cholesterol, LDL-C; low-density lipoprotein cholesterol, MCH; mean corpuscular hemoglobin, MCHC; mean corpuscular hemoglobin concentration, MCV; mean corpuscular volume, RBC; red blood cell, T-CHO; total cholesterol, WBC; white bold cell. Differences between the placebo and PLP groups were analyzed by using analysis of covariance (ANOVA).

**Table 3:** Results of two-factor analysis of variance (ANOVA) on morning systolic blood pressure (SBP), morning mean blood pressure (MBP), morning diastolic blood pressure (DBP), nocturnal SBP, nocturnal MBP, and nocturnal DBP data obtained in the placebo and *Perilla frutescens* leaf powder-intake subjects with home SBP ≥ 120. Data are presented in Figure 1. SBP, systolic blood pressure; MBP, mean blood pressure; DBP, diastolic blood pressure; PLP, *Perilla frutescens* leaf powder-intake group.

Placebo vs. PLP		Week	Group	Week × Group Interaction
**Morning SBP**	*p*-value	0.012	0.053	0.005
	F-value	11.26	1.56	2.02
**Morning MBP**	*p*-value	0.052	0.067	0.242
	F-value	6.12	1.50	1.52
**Morning DBP**	*p*-value	0.114	0.096	0.343
	F-value	3.25	1.43	1.10
**Nocturnal SBP**	*p*-value	0.398	<0.0001	0.016
	F-value	0.81	3.24	1.80
**Nocturnal MBP**	*p*-value	0.346	0.019	0.113
	F-value	0.96	2.78	1.28
**Nocturnal DBP**	*p*-value	0.144	0.013	0.170
	F-value	2.71	1.84	1.30

**Table 4:** Fatty acid profile (µg/mg protein) of the erythrocyte plasma membrane in the placebo and *Perilla frutescens* leaf powder-intake groups.

	Baseline		6-months	
	Placebo (n = 30)	PLP (n = 30)	Placebo (n = 25)	PLP (n = 28)
PLA	31.5 ± 1.7	34.1 ± 1.8	37.2 ± 1.0	37.9 ± 1.1
STA	24.9 ± 1.4	26.2 ± 1.4	29.9 ± 0.8	30.1 ± 0.9
OLA	18.6 ± 1.1	20.3 ± 1.1	23.8 ± 0.7	24.1 ± 0.7
LLA	13.9 ± 0.8	15.1 ± 0.9	17.7 ± 0.8	18.0 ± 0.9
ALA	0.37 ± 0.02	0.37 ± 0.02	0.26 ± 0.01	0.29 ± 0.01 *
AA	16.7 ± 1.1	17.5 ± 1.1	20.7 ± 0.8	21.1 ± 0.8
EPA	2.0 ± 0.2	2.4 ± 0.2	2.8 ± 0.3	2.8 ± 0.2
DPA	3.1 ± 0.2	3.3 ± 0.2	3.8 ± 0.1	3.9 ± 0.1
C24:0	8.3 ± 0.5	8.9 ± 0.5	9.9 ± 0.3	9.9 ± 0.3
DHA	11.1 ± 0.7	11.7 ± 0.8	12.7 ± 1.2	13.4 ± 0.6
C24:1	7.7 ± 0.5	8.0 ± 0.5	9.9 ± 0.3	9.9 ± 0.3
TFA	138.2 ± 7.8	147.8 ± 8.1	168.5 ± 4.7	171.3 ± 5.0

Values are expressed as the standard error of mean (SEM). Placebo: the placebo group, PLP, *Perilla frutescens* leaf powder-intake group. AA, arachidonic acid; DHA, docosahexaenoic acid; DPA, docosapentaenoic acid; EPA, eicosapentaenoic acid; LLA, linoleic acid; ALA, α-linolenic acid; OLA, oleic acid; PLA, palmitic acid; STA, stearic acid; TFA; total fatty acids. * *P* < 0.05 compared with the placebo group.

**Table 5:** Nutrient composition of pectin fiber.

Proximate Composition		Minerals	
Energy (kcal)	260.2	Sodium (mg)	1900
Protein (g)	1.0	Calcium (mg)	100
Fat (g)	0	Potassium (mg)	50
Carbohydrate (g)	38.1		
Fiber (g)	52.1		
Moisture (g)	5.7		
Ash (g)	3.2		

Nutritional values per 100 g of dry powder.

**Table 6:** Nutrient composition of the leaf powder of *Perilla frutescens* var. *frutescens* dried using a microwave under reduced pressure.

**Proximate Composition**		**Minerals**	
Energy (kcal)	323	Sodium (mg)	9.6
Protein (g)	22.0	Phosphorus (mg)	382
Fat (g)	7.4	Iron (mg)	45.4
Carbohydrate (g)	27.3	Calcium (g)	1.59
Fiber (g)	29.4	Potassium (g)	1.38
Moisture (g)	6.6	Magnesium (mg)	287
Ash (g)	7.3	Zinc (mg)	4.69
**Vitamins**		**Amino acids**	
α-carotene (mg)	0.1	l-Lysine (g)	1.34
β-carotene (mg)	24.3	l-Histidine (g)	0.53
Thiamine (mg)	0.53	l-Phenylalanine (g)	1.21
Riboflavin (mg)	0.86	l-Leucine (g)	1.89
Vitamin B_6_ (mg)	1.09	l-Isoleicine (g)	0.93
Vitamin B_12_ (mg)	N.D.	l-Methionine (g)	0.41
Vitamin C (mg)	6	l-Valine (g)	1.23
Vitamin E (mg)	38.8	l-Threonine (g)	1.02
Vitamin K_1_ (mg)	4.86	l-Tryptophan (g)	0.42
Vitamin K_2_ (mg)	N.D.	GABA (mg)	6
**Fatty acids**		**Others**	
Palmitic acid (g)	0.61	Lutein (mg)	44.9
Stearic acid (g)	0.09	Chlorophyll (mg)	574
Oleic acid (g)	0.10	Rosmarinic acid (g)	2.3
Linoleic acid (g)	0.47		
α-Linolenic acid (g)	2.23		

Nutritional values per 100 g of dry powder. GABA, γ-aminobutyric acid; N.D., not detectable. Nutritional composition except the fatty acids was measured in Japan Food Research Laboratories (Tokyo). Fatty acid composition was analyzed by gas chromatography as shown in the methods.
